# Mapping the metabolism of five amino acids in bloodstream form *Trypanosoma brucei* using U-^13^C-labelled substrates and LC–MS

**DOI:** 10.1042/BSR20181601

**Published:** 2019-05-17

**Authors:** Katharina Johnston, Dong-Hyun Kim, Eduard J. Kerkhoven, Richard Burchmore, Michael P. Barrett, Fiona Achcar

**Affiliations:** 1 Welcome Centre for Integrative Parasitology, Institute of Infection, Immunity & Inflammation, University of Glasgow, Glasgow, UK; 2 Centre for Analytical Bioscience, School of Pharmacy, University of Nottingham, Nottingham, UK; 3 Systems and Synthetic Biology, Department of Biology and Biological Engineering, Chalmers University of Technology, Göteborg, Sweden

**Keywords:** amino acid metabolism, mass spectrometry, parasitic protozoa, trypanosomes

## Abstract

The metabolism of the parasite *Trypanosoma brucei* has been the focus of numerous studies since the 1940s. Recently it was shown, using metabolomics coupled with heavy-atom isotope labelled glucose, that the metabolism of the bloodstream form parasite is more complex than previously thought. The present study also raised a number of questions regarding the origin of several metabolites, for example succinate, only a proportion of which derives from glucose. In order to answer some of these questions and explore the metabolism of bloodstream form *T. brucei* in more depth we followed the fate of five heavy labelled amino acids – glutamine, proline, methionine, cysteine and arginine – using an LC–MS based metabolomics approach. We found that some of these amino acids have roles beyond those previously thought and we have tentatively identified some unexpected metabolites which need to be confirmed and their function determined.

## Introduction

*Trypanosoma brucei* is the causative agent of human and animal African trypanosomiasis. It is a protozoan parasite, transmitted to its mammalian host via a tsetse fly vector. In both hosts the parasite is extracellular, proliferating in the mammalian bloodstream and central nervous system and the tsetse fly midgut from where it migrates through different body tissues before reaching the salivary gland for transmission. *T. brucei* metabolism is adapted to these disparate environments and significant differences distinguish its metabolism in the different forms of the parasite [1,2].

Within the fly, the main carbon and energy source of the procyclic form trypanosomes is proline, while the bloodstream form parasite uses glucose fed glycolysis as its main energy source [3]. Because of the very high rate of glycolysis, it had long been accepted that the glucose consumed by the parasite was essentially excreted as pyruvate and glycerol. However, the availability of high throughput metabolomics technology, in conjunction with use of isotope labelled substrates, has permitted extensive tracing of glucose metabolism in both the procyclic form [3] and bloodstream form [5] organisms. In bloodstream forms it is clear that glucose enters a plethora of pathways beyond simple glycolysis [5], as is also true for the procyclic form. Unexpected metabolites such as octulose 8-phosphate were identified and labelling patterns revealed a significant role for a succinate shunt generating partially oxidised end products of metabolism. Interestingly, only a proportion of some of these end products, including succinate, is derived from glucose, implying that other sources are used by the cell in their production. It has also been shown in *in vitro* culture that bloodstream form organisms consume substantial quantities of threonine [6,7] and that a supply of either the aromatic amino acids (tryptophan, tyrosine and phenylalanine) or glutamine was necessary for growth [6]. These appear to be critical cellular sources of nitrogen, fixed via aminotransferase actions. Intriguingly, indole pyruvate, the product of tryptophan has possible immunomodulatory roles [8].

Amino acids clearly play key roles in many aspects of trypanosome metabolism. For example, the unusual metabolite trypanothione (*N*^1^,*N*^8^-bis(glutathionyl)-spermidine) derives from glutathione (itself consists of the glutamate, cysteine, glycine tripeptide) and the polyamine spermidine, which derives from ornithine via putrescine through sequential addition of aminopropyl groups from decarboxylated *S*-adenosylmethionine, itself a product of methionine and adenosine. In order to learn more about the utilisation of different amino acids across the trypanosome’s metabolic network we have investigated the distribution of atoms derived from glutamine, proline, methionine, cysteine and arginine using heavy labelled precursors and LC–MS, as these five amino acids will address specific gaps in our current metabolic map as detailed below.

## Materials and Methods

### Parasite culture and Δ*argk* generation

*T. brucei brucei* bloodstream form (s427) was cultured *in vitro* in Creek’s ‘Minimal’ Media (CMM) [6] with 10% FBS Gold at 37°C, 5% CO_2_. For continuous growth, cell densities were kept between 5 × 10^4^ and 2 × 10^6^ cells/ml and the cell density was checked every 48 h using an improved Neubauer haemocytometer. For growth curves, the cells were counted every 24 h and cell counts performed in triplicate.

The three arginine kinase genes, Tb427tmp.160.4560, Tb427tmp.160.4570, Tb427tmp.160.4590 (Tb927.9.6290, Tb927.9.6250 and Tb927.9.6210 in the 927 reference genome) were knocked out in the bloodstream form s427 strain to obtain strain *Δak*. The three isoforms are located in an array in the genome (Supplementary Figure S1A), interspaced by a putative gene with unknown function Tb427tmp.160.4580 (Tb927.9.6270). A strategy was designed where the region covering all four genes was replaced with antibiotic resistance cassettes (Supplementary Figure S1B). The 5′ and 3′ flanking regions were amplified from genomic DNA (all primers are detailed in Supplementary Table S1) and cloned flanking a hygromycin or puromycin resistance cassette in vector pTBT [9]. Parasites were transformed in two rounds with NotI-linearised constructs as described previously [10], to replace both allelic regions with the hygromycin and puromycin resistance cassettes through homologous recombination. Positive clones were selected with 2.5 μg/ml hygromycin and 0.2 μg/ml puromycin, and correct integration of the resistance cassettes was confirmed by PCR (Supplementary Figure S1C). Absence of additional isoforms of TbAK in the *Δak* strain was validated by PCR, following standard procedures [11] and probed with Easytides ^32^P-ATP (Perkin Elmer) incorporated into TbAK using the Stratagene Prime-it kit.

### Intracellular metabolite extraction from parasites

Trypanosomes were grown to mid-log phase and a sample volume equivalent to 5 × 10^7^ cells was rapidly cooled to 4°C by submerging a 50 ml Falcon tube containing the parasites in a dry ice/ethanol bath. Samples were kept on ice (or 4°C) from this step onwards. Samples were centrifuged at 1250***g*** for 10 min, and most supernatant, except 1 ml, was removed. The pellet was resuspended in the remaining 1 ml of medium. This was transferred to an Eppendorf tube and briefly centrifuged at 1250***g*** to completely remove the supernatant. The cell pellet was washed in cold 1× PBS and metabolites extracted by resuspending in 100 μl chloroform:methanol:water (ratio1:3:1 v/v/v) with internal standards and by vigorously shaking for 1 h at 4°C. Samples were centrifuged at 16,000***g*** for 10 min and the supernatant collected and stored at −80°C under argon.

### ^13^C-labelled tracking

*T. b. brucei* strain 427 was grown in CMM + 10% FBS Gold (PAA, Piscataway, NJ) at 37°C, 5% CO_2_ for U-^13^C-labelled tracking. For l-methionine studies, a 1:1 mix of unlabelled and U-^13^C l-methionine (50 μM l-methionine and 50 μM U-^13^C l-methionine) was added to the parasite culture medium (starting density of 2 × 10^4^ cells/ml). For l-glutamine and l-cysteine studies, a 1:1 mix of unlabelled and U-^13^C glutamine or cysteine (500 μM unlabelled and 500 μM U-^13^C) was added to the parasite culture medium. For l-proline and l-arginine, 100% labelled compound was used at a concentration of 200 μM as significant levels of unlabelled l-proline and l-arginine are present in the serum. Cells were incubated at 37°C, 5% CO_2_ for 48 h, and metabolites extracted as described above. Samples were prepared in triplicate (biological replicates) and for every labelled setup a control with the equal concentration of unlabelled compound was set up, also in triplicate. Fresh medium and spent medium controls (for the l-methionine, l-proline and l-arginine experiments) were also collected and 5 μl were added to 100 μl extraction solvent (CMW 1:3:1) prior to analysis.

Labelled compounds were obtained from Cambridge Isotope Laboratory, Inc.: (l-methionine, ^13^C5, enrichment 99%, cat: CLM-893-H-0.1; l-proline, ^13^C5, enrichment 99%, cat: CLM-2260-H; l-arginine, ^13^C6, enrichment 99%, cat: CLM-2265-H-0.1; l-cysteine, ^13^C3, enrichment 99%, cat: CLM-4320-H-PK; l-glutamine, ^13^C5, enrichment 99%, cat: CLM-1822-H-PK).

### Sample preparation for pH stress

*T. b. brucei* strain 427 wild type (WT) and arginine kinase knockout (*Δak*) cells were cultured in CMM with 10% FBS Gold at 37°C, 5% CO_2_ as described above and allow them to reach a density of 2 × 10^6^ cells/ml. After a density of 2 × 10^6^ cells/ml was achieved, WT and *Δak* cells were centrifuged at 1250***g*** for 10 min and the cells were then resuspended in CMM at pH 8.7 (pH stress) and pH 7.4 (controls) and incubated for 120 min.

The cells were then extracted as described in the section ‘Intracellular metabolite extraction from parasites’. Equal volumes from all the samples were mixed to create a quality control (QC) sample in order to assess instrument performance. All samples were prepared in four biological replicates.

### Liquid chromatography–mass spectrometry

The sample platform chosen for this project was liquid chromatography coupled with mass spectrometry. All samples were separated on a Dionex Ultimate 3000 RSLC liquid chromatography system (Dionex, Camberley, Surrey) with ZIC-pHILIC 150 × 4.6 mm, 5 µm column (Merck Sequant) prior to mass detection on an Q-Exactive Orbitrap mass spectrometer (Thermo Fisher Scientific, Hemel Hempstead, U.K.). LC–MS analysis was performed in positive and negative electrospray mode (*m/z* 70–1400, resolution 50,000), using 10 μl injection volume and a flow rate of 300 μl/min. For HPLC gradient, ZIC-pHILIC solvent A was 20 mM ammonium carbonate in H_2_O, and solvent B was 100% acetonitrile. A linear gradient of the mobile phases was applied, in which samples were eluted from 80% B to 5% B over 15 min, followed by another linear gradient from 5% B to 80% B over 2 min, and 80% B was held for 7 min for re-equilibration. With each experiment a set of authentic standards was run prior to the sample set.

### Data processing and analysis

Raw LC–MS data files were converted to mzXML using ProteoWizard msconvert [12]. Data were processed using XCMS [13] for peak picking and mzMatch for peak grouping, filtering and putative identifications [14]. Metabolite identification was performed by matching accurate masses and retention times of authentic standards. Level 1 metabolite identification according to the metabolomics standards initiative [15,16], but when standards were not available masses and labelling patterns were used, hence these identifications should be considered as putative (Level 2 identification). Isotopic profiles were analysed using mzMatch-ISO [4].

In order to visualise metabolic differences between control and high pH treated cells, orthogonal partial least squares-discriminant analysis (OPLS-DA) was carried out by SIMCA-P 14.1 (Umetrics AB, Umea, Sweden). Mass ions (*m/z*) that were changed significantly between the two groups were selected by variable importance in projection values (VIP) where the values greater than one were determined as potential key markers. Additionally, univariate *t*-test analysis was performed using a web-based analysis tool, Metaboanalyst [17] to cross check significant difference between control and treated cells.

The mzXML files of all the experiments described in the present study are available in the Metabolights database at https://www.ebi.ac.uk/metabolights/MTBLS624.

## Results and discussion

A recent study by Creek et al. [5] showed that glucose was a source of carbon for many pathways beyond glycolysis. The present study also raised new questions, as many of these pathways only use glucose for part of their carbon needs. To extend the measured metabolic map of bloodstream form *T. brucei*, we followed the fate of five key amino acids, arginine, cysteine, glutamine, methionine and proline inside the cells, using U-^13^C-labelled amino acids and LC–MS.

### Glutamine

As previously reported [6,7,18], glutamine is consumed by bloodstream form *T. brucei* from CMM in large quantities where it has been proposed it mainly serves as a key amino group donor. 2-Oxoglutarate and 2-oxoglutaramate as well as some glutamate are secreted into the medium as bloodstream forms grow [6,18]. Furthermore, significant quantities of succinate are secreted by bloodstream forms [6,7,18], of which only 52% comes from glucose via the essential phosphoenolpyruvate carboxykinase [5]. The remaining succinate must be produced from another carbon source, and glutamine-derived 2-oxoglutarate is a likely candidate. To test this hypothesis, we incubated *T. brucei* bloodstream form for 48 h in CMM with 50% U-^13^C glutamine and then analysed the intracellular metabolome using LC–MS (see Materials and Methods).

**Figure 1:**
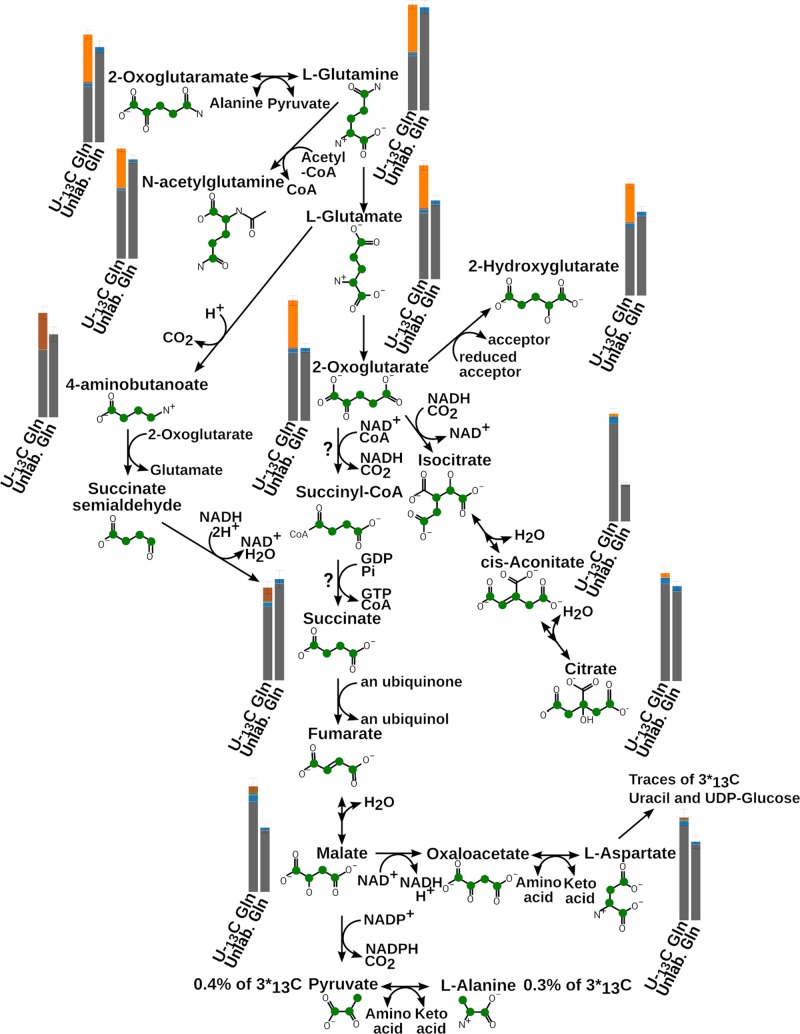
Glutamine labelled experiments results The bar charts show the measured proportions of the different number of ^13^C: grey represents fully unlabelled metabolite, blue 1 carbon ^13^C, brown 4 carbons ^13^C and orange 5 carbons ^13^C. The green dots on the molecules represent the theoretical individual ^13^C starting with a fully labelled precursor following this pathway.

**Figure 2:**
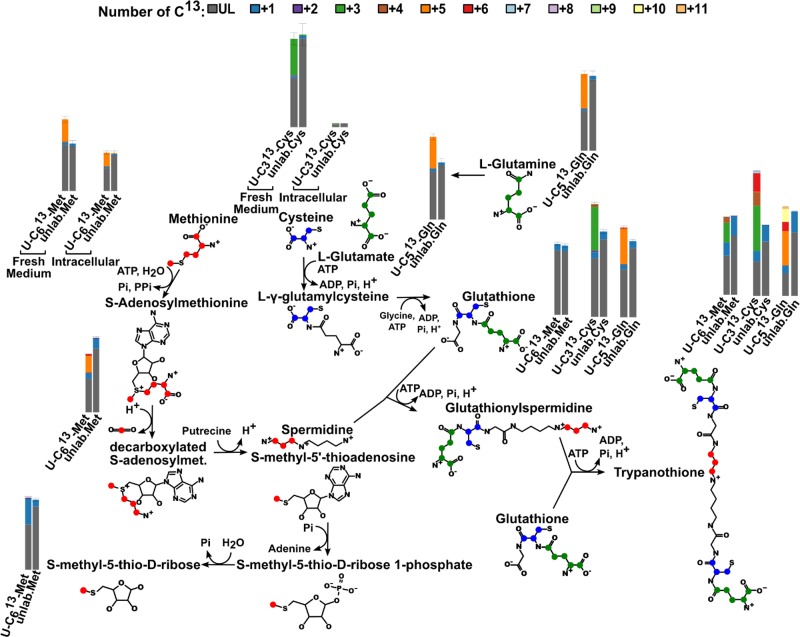
Trypanothione biosynthesis pathway as seen using 50% U-^13^C glutamine, cysteine or methionine The bar charts show the measured proportions of the different number of ^13^C (colour code is shown at the top of the figure). The green dots on the molecules represent the theoretical individual ^13^C coming from fully labelled glutamine, blue carbons come from fully labelled cysteine and red from fully labelled methionine.

The results (see Figure 1) reveal that 45% of intracellular glutamine is 5 carbon labelled (additional unlabelled glutamine from the serum likely having a small dilution effect). Acetylated glutamine is also present (*N*-acetylglutamine labelled at 37 ± 8%). Glutamate is 37.5 ± 0.3% fully labelled and 2-oxoglutarate 39.0 ± 0.3%; confirming that the primary source of glutamate and 2-oxoglutarate inside the cell is medium-derived glutamine. Labelling can also be seen in glutathione (39.4 ± 0.1%) and trypanothione disulphide (39 ± 1%), as expected (see Figure 2).

Although a canonical glutaminase (EC 3.5.1.2) encoding gene cannot be found in the *T. brucei* genome [19], several reactions could be responsible for producing glutamate from glutamine, for example: (i) a GMP synthase (EC. 6.3.5.2, gene Tb927.7.2100) that converts XMP to GMP uses glutamine as an amino group donor [20], (ii) a glutamine-dependent carbamoyl-phosphate synthase (EC 6.3.5.5, putative gene Tb927.5.3800, syntenic orthologue of the identified *Leishmania mexicana* and *Trypanosoma cruzi* genes [21] also uses glutamine as an amino group donor to produce carbamoyl-phosphate from hydrogen carbonate [22] and is also essential [23], (iii) an asparagine synthase (Tb927.7.1110) can use either ammonia (EC 6.3.1.1) or glutamine to produce asparagine from aspartate [24].

Many reactions can produce 2-oxoglutarate from glutamate. For example, in *T. brucei*, glutamate dehydrogenase [25]. Two isoforms of aspartate aminotransferase (EC 2.6.1.1, mitochondrial isoform gene Tb927.11.5090, cytosolic isoform gene Tb927.10.3660) are also present in bloodstream forms [27] and alanine aminotransferase (EC 2.6.1.2, gene Tb927.1.3950) is expressed at high levels [28]. The putatively identified 2-oxoglutaramate is present in the labelled form at high levels (43.5 ± 0.1%) and is probably derived from the glutamine aminotransferase (EC 2.6.1.15, gene Tb927.10.11970) that produces 2-oxoglutaramate and alanine from glutamine and pyruvate [28].

The total succinate pool is present with 14.9 ± 0.1% containing 4 labelled carbons indicating that part of the total succinate pool is derived from glutamine, most likely via 2-oxoglutarate. Indeed, enzyme activities of 2-oxoglutarate dehydrogenase (EC 1.2.4.2) and acetyl:succinate CoA-transferase (EC 2.8.3.18, gene Tb927.11.2690) have been measured in bloodstream forms, albeit at lower levels than in procyclics [29,30]. In addition to succinate, 4 carbon labelled malate is also present (6.2 ± 0.1% of the total measured malate) presumably due to a reverse flux from succinate [7]. Malate then contributes to the low levels of labelled aspartate (2.6 ± 0.2% of the total) via malate dehydrogenase (EC 1.1.1.299, cytosolic isoform gene Tb927.11.11250) [29,31,32] and aspartate aminotransferase. The promiscuous action of malate dehydrogenase on 2-oxoglutarate [33] also produces 5 carbon labelled 2-hydroxyglutarate [34] (34.1 ± 0.5% of the total) which is transformed to 2-oxoglutarate by 2-hydroxyglutarate dehydrogenase (EC 1.1.99.2, putatively annotated gene Tb927.10.9360).

Bloodstream form *T. brucei* contain large concentrations of pyruvate and alanine in their cytoplasm [18,35], most of which is derived from glucose (close to 100% in [5], although labelling experiments show that a small but significant quantity of the total (0.4 ± 0.02% of the pyruvate and 0.3±0.03% of the alanine) is labelled from glutamine (see Figure 4). Malic enzyme (EC 1.1.1.40, mitochondrial isoform Tb927.11.5450, cytosolic isoform Tb927.11.5440), the activity of which has been measured in bloodstream forms [29,33,36,37] could produce this pyruvate and subsequently alanine via alanine aminotransferase and glutamine aminotransferase.

Small amounts of 5 carbon labelled citrate/isocitrate (these two isomers cannot be distinguished on this platform) and cis-aconitate (4.0 ± 0.6% of citrate and less than 1% of cis-aconitate) also derive from the labelled glutamate, as also seen from labelled glucose (approx. 11% of citrate/isocitrate is found labelled from glucose and 5% of *cis*-aconitate) [5]. Isocitrate dehydrogenase can produce isocitrate from 2-oxoglutarate (EC 1.1.1.42, gene Tb927.11.900) and the protein is identified in bloodstream forms [33,37,38] despite its activity being below the detection threshold when assayed [30]. Aconitase activity was detectable [30] and the protein detected [39] (EC 4.2.1.3, gene Tb927.10.14000), thus explaining the labelled cis-aconitate and citrate. The proportion of the labelled derivatives is low when compared with unlabelled due to the parasites accumulating large quantities of citrate from medium [38]. It is clear that glutamine can be used to fuel various pathways in bloodstream form *T. brucei*, with a primary role in provision of amino groups to the nitrogen pool of the parasites.

### Proline

Proline is the primary source of carbon and energy in procyclic form trypanosomes and critical to viability in the tsetse fly [40]. Enzymes involved in its catabolism are substantially less abundant in the bloodstream form and it has been assumed that proline has little metabolic use to these cells [41]. Given that the presence of peptidases in the medium increases the concentration of unlabelled proline over time [18], the cells were incubated in medium containing 200 µM proline 100% U-^13^C-labelled. After 48 h 49 ± 1% of the proline inside cells was labelled (unlabelled proline coming from the serum causes the dilution) while very small quantities (less than 1%) of succinate, malate, 2-oxoglutarate and glutamate were labelled (4 carbons for succinate and malate, 5 for glutamate and 2-oxoglutarate) (see Supplementary File S4) confirming that these cells barely utilise l-proline through catabolic pathways although they maintain low level activity of the enzymes necessary to do so.

### Methionine

Methionine has several roles of fundamental importance in trypanosomes. For example, it is the primary source of methyl groups in a multitude of reactions when converted to *S*-adenosylmethionine (AdoMet) and also of aminopropyl groups in growing polyamine chains after production of the decarboxylated form. Creek et al. previously revealed that *S*-adenosyl-l-methionine is 5 carbon labelled when using U-^13^C glucose due to labelling of the ribose moiety of the adenosyl group (76% labelled in [5]. Decarboxylated Adomet is used exclusively for the conversion of putrescine to spermidine which creates *S*-methyl-5′-thioadenosine (MTA) once the aminopropyl group has been transferred to the growing polyamine chain. In many cell types, MTA is recycled to methionine and this was presumed to occur also for bloodstream form *T. brucei* [42]. However, Creek et al. showed this is not the case. Labelling with 50% U-^13^C l-methionine and identifying its metabolic products both within the parasites and their medium has given more insight into the fate of MTA, whose accumulation is generally toxic [43,44].

**Figure 3:**
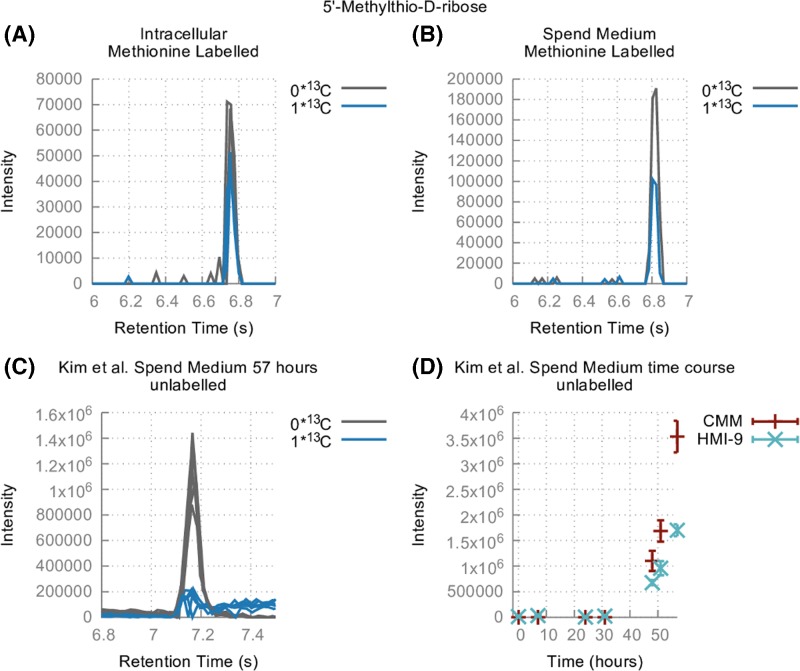
Putative methylthio-d-ribose in the U-^13^C methionine experiment (**A and B**) and in Kim et al. [18] spend medium experiment (**C and D**)

MTA is not converted to l-methionine (see Figure 2) using the classical methionine cycle since this pathway would create 1 carbon labelled l-methionine from U-^13^C l-methionine and no excess 1 carbon labelled product is detected when compared with the unlabelled control. 35 ± 6% of intracellular methionine is labelled with 5 carbons for 48 h and 28.2 ± 0.1% of *S*-adenosyl-l-methionine is also 5 carbon labelled. A peak corresponding to the mass of MTA was labelled with a single carbon (36 ± 2% 1 carbon ^13^C-labelled). This peak is only detected within cells and not in the spent medium. By contrast, a low intensity peak with a significant proportion of 1 carbon label corresponding to the mass of methylthioribose was identified in spent medium (see Figure 3A,B). Since this peak was of low intensity we investigated its appearance in spent medium in a time course experiment [18]. This metabolite accumulates in the medium proportionally to the cell density (see Figure 3C,D). This indicates that bloodstream form *T. brucei* metabolise methionine via AdoMet to MTA which is converted to methylthioribose 1-phosphate (EC 2.4.2.28, gene Tb927.7.7040) [45], then dephosphorylated to methylthioribose which is secreted from the cell. It appears likely, therefore, that *T. brucei* has evolved a mechanism to detoxify MTA generated during polyamine biosynthesis by converting to MTR which is subsequently secreted from the cell, as is also the case in *E. coli* [46]*.*

**Figure 4:**
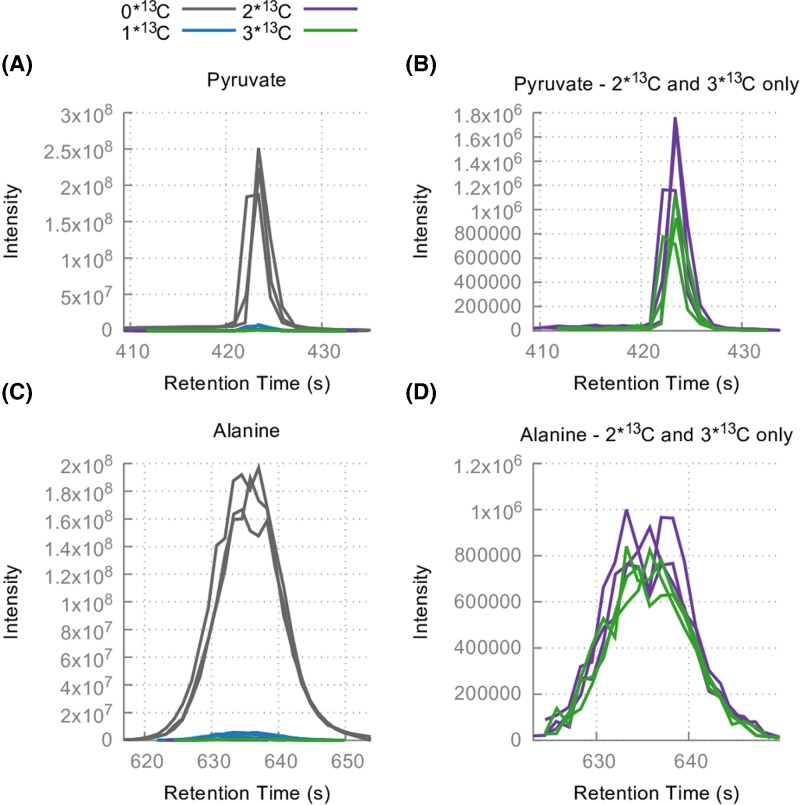
Pyruvate (**A and B**) and alanine (**C and D**) as seen in the 50% U-^13^C glutamine experiment

A metabolite with mass consistent with methionine’s ketoacid derivative, 2-oxo-4-methylthiobutanoic acid, was also identified. This metabolite is present with 33 ± 1% 5 carbon labelled both intracellularly and in the spent medium, indicating it arises from the transamination of methionine and not MTA recycling (in which case it would be 1 carbon ^13^C-labelled). This transamination is probably catalysed by aspartate aminotransferase [26].

During spermidine synthesis, three carbons in the aminopropyl group released from decarboxylated AdoMet are labelled and since spermidine is then incorporated into trypanothione we identify 3 carbon labelled trypanothione (see Figure 2).

**Figure 5:**
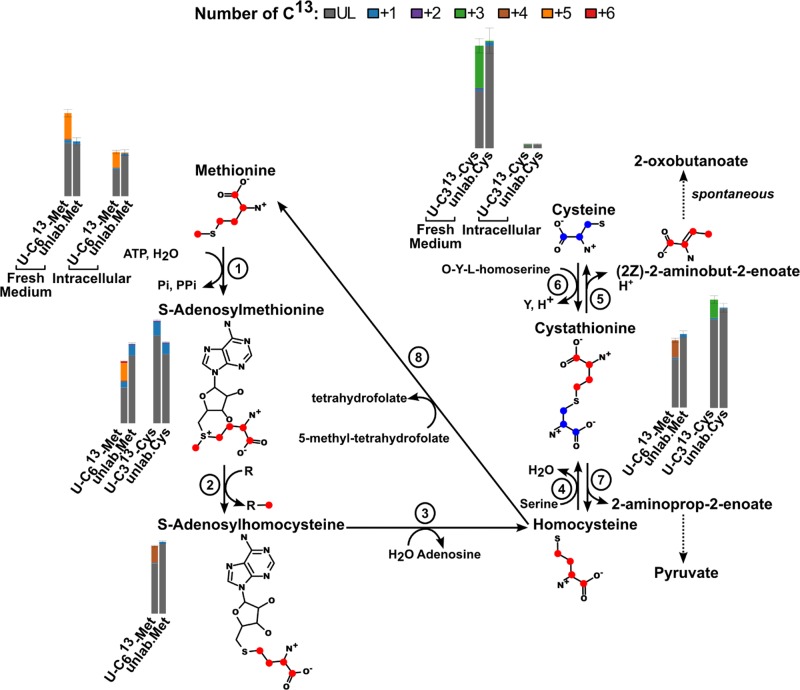
*S*-Adenosylhomocysteine recycling pathway The bar charts show the measured proportions of the different number of ^13^C (colour code is shown at the top of the figure). The blue dots on the molecules represent the theoretical individual ^13^C coming from cysteine and red from methionine.

*S*-Adenosyl-l-methionine is also a major methyl group donor. Methylation reactions convert *S*-adenosyl-l-methionine into *S*-adenosyl-l-homocysteine, which we detect with 24 ± 4% 4 carbon labelled (see Figure 5). Several metabolites can also be detected as methylated derivatives in this experiment (Table 1 gives a list of the labelled metabolites detected, the number of labelled carbons and their potential functions).

**Table 1:** Methylated metabolites detected in the U-^13^C methionine experiment

Metabolite	Source of identification	Carbons ^13^C-labelled	Putative function	Putative gene involved	Reference
*N*γ-Monomethyl-l-arginine	Mass and labelling	1	Protein/histone methylation	Tb927.1.4690 (type I) Tb927.5.3960 (type I) Tb927.10.640 (type II) Tb927.7.5490 (type III) Tb927.10.3560	[48,50] – five putative arginine protein methyltransferases: type I: [51,52], type II: [53], type III: [54] (histone: [55,56])
*N*ω, *N*ω′-Dimethyl-l-arginine	Mass and labelling	1 and 2	Protein/histone methylation	Tb927.1.4690 (type I) Tb927.5.3960 (type I) Tb927.10.640 (type II) Tb927.10.3560	See *N*γ-monomethyl-l-arginine
*N*6-Methyl-l-lysine	Mass and labelling	1	Protein/histone methylation	Tb927.8.1920, Tb927.1.570	[59–62]
*N*6,*N*6,*N*6-Trimethyl-l-lysine	Mass and labelling	1, 2 and 3	Protein/histone methylation	Tb927.8.1920, Tb927.1.570	See *N*6-methyl-l-lysine
Methylguanine	Mass and labelling	1	RNA cap	Tb927.11.4890, Tb927.10.7940	[59,60]
*N*α-Methylhistidine	Mass, retention time (match standard) and labelling	1	Histidine methylation seen in histones and other proteins in *Leishmania*		[61] (*Leishmania*)

The product of these methylation reactions, *S*-adenosyl-l-homocysteine is another toxic thiol which needs to be removed from the system [47]. The classical pathway of detoxification involves a salvage pathway that ultimately produces l-cysteine (see Figure 5). Labelled carbons from the methionine precursor are first passed on to homocysteine (undetected) via adenosylhomocysteinase (EC 3.3.1.1, putative gene Tb927.11.9590 [48]), then cystathionine via cystathionine β-synthase (EC 4.2.1.22, putative genes Tb11.02.5400b and Tb11.02.5400 – orthologue of the identified *T. cruzi* gene [49]) and finally 2-aminobut-2-enoate via cystathionine γ-lyase (EC 4.4.1.1, putative genes Tb927.9.12320 and Tb11.v5.0869, orthologues of the identified *Leishmania major* gene [50] and oxobutanoate). Of these four metabolites, we detect cystathionine which is indeed 26 ± 1% 4 carbon labelled. This supports the presence of this salvage pathway as previously indicated by Bacchi et al. using ^35^S labelled methionine [51]. However, since bloodstream form *T. brucei* consume large quantities of cysteine from their medium [6,52] the salvage pathway is probably more important in preventing accumulation of toxic *S*-adenosyl-l-homocysteine and l-homocysteine than in providing cysteine which needs to be present in the medium for the parasite to be able to grow [52].

Homocysteine can also be reconverted to methionine by 5-methyltetrahydropteroyltriglutamate-homocysteine *S*-methyltransferase (EC 2.1.1.14, putative gene Tb927.8.2610) or homocysteine *S*-methyltransferase (EC 2.1.1.10, putative gene Tb927.1.1270) [53]. The first reaction would produce four carbons ^13^C-labelled methionine. However, whilst a metabolite of this mass is detected, it is also found at the same level in fresh medium containing U-^13^C methionine, indicating that it is a contaminant of the label stock and not a product of cellular metabolism. The second reaction, homocysteine *S*-methyltransferase [53], would produce U-^13^C methionine if the co-substrate *S*-methyl-l-methionine is also derived from *S*-adenosyl-l-methionine. However, this pathway would also produce additional *S*-adenosyl-l-homocysteine and therefore would not reduce the concentration of this metabolite.

This data indicate that methionine is a key donor of methyl groups following conversion to AdoMet and aminopropyl groups in polyamine biosynthesis. In each case, potentially toxic by-products are produced, and it appears that the parasite has evolved a mechanism to detoxify MTA, by conversion in two steps to MTR which is secreted, and *S*-adenosyl-l-homocysteine through conversion to cysteine.

### Cysteine

26 ± 1% of cystathionine is labelled when U-^13^C methionine is added to culture medium. As 35 ± 6% of total intracellular methionine is labelled after 48 h, approx. 74% of cystathionine would appear to come from the *S*-adenosyl-l-homocysteine recycling pathway. This indicates that one or more other pathways must account for the remaining 26%. The most likely additional source of cystathionine is cysteine, which is consumed in large quantities by bloodstream form *T. brucei* [6]. Cells were grown for 48 h in CMM containing 50:50 U-^13^C cysteine: unlabelled cysteine to follow the fate of cysteine inside the cells.

Labelled cysteine is seen within cells, but the peaks are of too low intensity to allow quantification of relative proportions of labelled and unlabelled versions. Around 20% of cystathionine is labelled at three carbons, indicating it is derived either from the reverse reaction of cystathionine γ-lyase (Figure 5, reaction 5) or from cystathionine γ-synthase (Figure 5, reaction 6) [54]. No orthologues of known variants of the latter enzyme are found in the genome although the presence of a non-canonical enzyme cannot be excluded. Cysteine-derived carbons could be transferred to 2-aminoprop-2-enoate by a cystathionine β-lyase (EC 4.4.1.8, protein sequence very similar to cystathionine γ-lyase) [54]. 2-aminoprop-2-enoate would in turn be converted to pyruvate. However, 2-aminoprop-2-enoate cannot be detected and we cannot see any trace of labelled pyruvate derived from cysteine in this dataset making its presence unlikely. Only growing cells in the presence of ^34^S labelled cysteine would allow us to prove or disprove this hypothesis.

**Figure 6:**
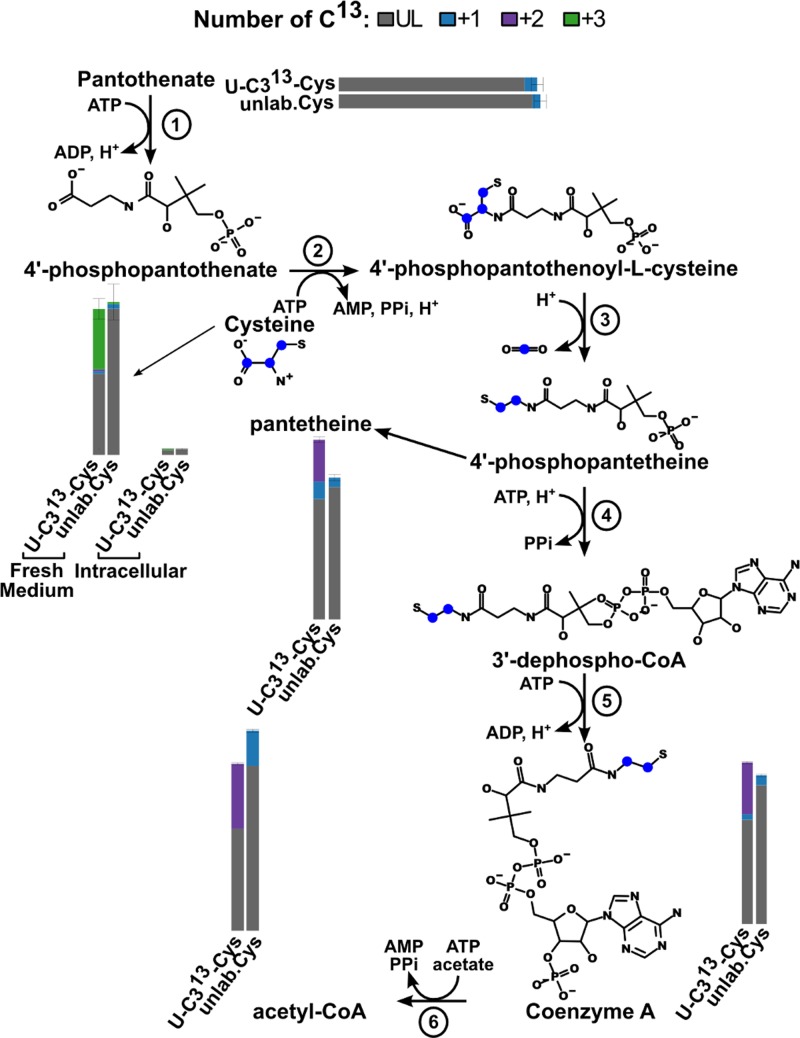
Coenzyme A biosynthesis pathway The bar charts show the measured proportions of the different number of ^13^C (colour code is shown at the top of the figure). The blue dots on the molecules represent the theoretical individual ^13^C coming from cysteine.

Cysteine is also essential to produce glutathione, trypanothione and coenzyme A (CoA). As expected, glutathione is present with 39 ± 4% 3 carbon ^13^C-labelled and trypanothione with 38 ± 5% 3 carbon ^13^C-labelled and 16 ± 2% 6 carbon ^13^C-labelled (see Figure 2). CoA is most likely produced from pantothenate taken up from the medium (see Figure 6), even though its consumption rate could not be measured due to the small amount likely required by the cells [18]. Indeed, we can detect coenzyme A and acetyl-CoA with 2 carbon labelled as the biosynthesis pathway predicts. We also detect a 2 carbon labelled metabolite the mass of which is consistent with pantetheine, a likely fragment of phosphopantetheine. The first four reactions of CoA synthesis are catalysed by enzymes that are all putatively annotated in the *T. brucei* genome but have never been tested (see Figure 6). The fifth reaction, dephospho-CoA kinase (EC 2.7.1.24, putative gene Tb927.6.710) has been localised to glycosomes [45]. Coenzyme A can then be converted to acetyl-CoA either via the pyruvate dehydrogenase complex (EC 1.2.1.-) or via the acetyl-CoA synthetase (EC 6.2.1.1, Tb927.8.2520 [55]) as shown by Mazet et al. [7].

### Arginine

Arginine plays several important roles in cellular physiology in many systems. It is, for example, a precursor to polyamine synthesis in most systems, producing ornithine via arginase. Furthermore, it generates citrulline and nitric oxide via the nitric oxide synthase (NOS). A gene encoding an enzyme of the arginase family was found in *T. brucei* [56] but the protein lacked arginase activity [57]. Instead it seems that ornithine is accumulated from outside the cells [58]. Here we investigated the fate of arginine in trypanosomes by growing them in presence of 200 µM U-^13^C arginine in CMM.

**Figure 7:**
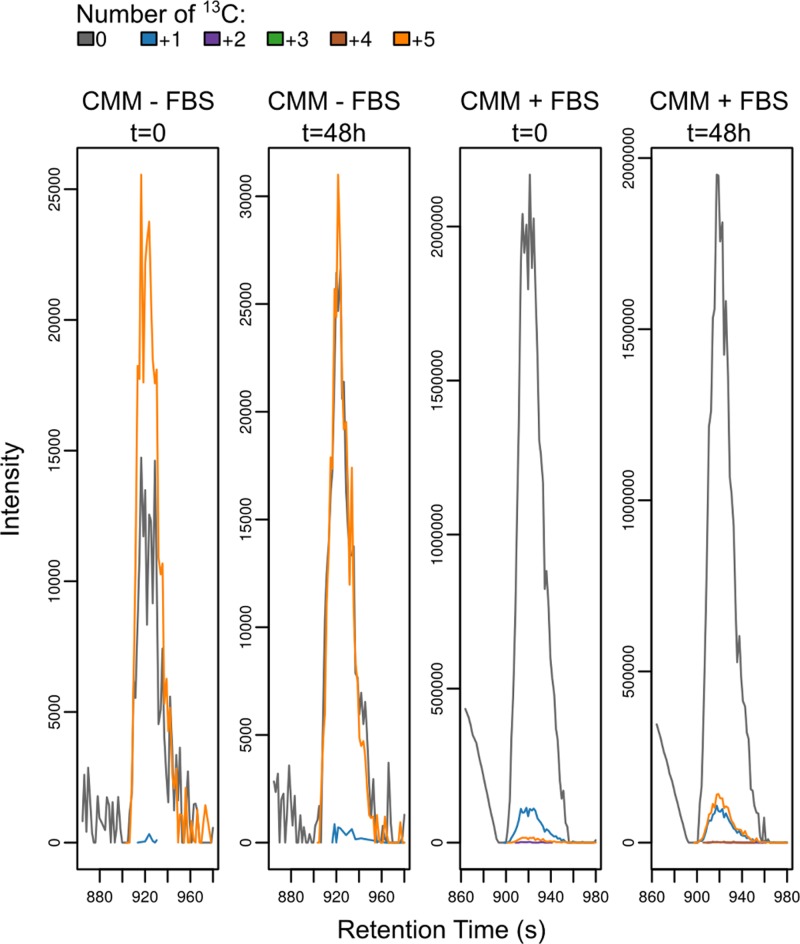
Peak of ornithine as seen in the medium (CMM) when arginine is fully labelled, with or without FBS but no cells (Intensity of the peak shown as a function of retention time).

By the time a steady state was reached, 40 ± 1% of intracellular arginine is fully labelled (a dilution effect is most likely caused by the peptidases secreted in the medium [18] freeing additional unlabelled arginine from serum proteins). 11.2 ± 0.5% of intracellular ornithine was labelled with 5 ^13^C carbons, consistent with it being produced by arginase, previously reported as missing from trypanosomes [57]. However, the same proportion of labelled ornithine can be seen in the spent medium (but not in the fresh medium), raising the possibility that some arginine is converted to ornithine in the medium, possibly by arginase in the serum. To test this hypothesis, we added 200 µM of U-^13^C arginine to the medium with or without FBS and without adding cells. After 48 h we can see an increase in the labelled ornithine produced only with added FBS. This reveals that ornithine is indeed produced from arginine when no cells are present (see Figure 7) hence the labelled ornithine observed inside trypanosomes appears to be created by arginase present in serum and then secondarily accumulated by trypanosomes.

The methionine labelling experiment revealed mono and dimethylarginine present with 22 ± 2% and 26 ± 1% of their total being labelled with 6 carbons. A small proportion of fully labelled citrulline (2.8 ± 0.7%, the fragmentation of this peak was checked and is consistent with citrulline) is also detected in cells but very little in the spent medium. Since *T. brucei* does not have NOS activity [59] it is likely that the citrulline derives from dimethylarginine via dimethylarginase activity although the canonical enzyme is not found in the genome (dimethylated arginines are found on histones for example).

A metabolite with a mass corresponding to 5-guanidino-2-oxo-pentanoate is also detected (37 ± 1% of its total quantity labelled). This metabolite would be produced by an aminotransferase reaction using arginine as the nitrogen donor.

**Figure 8:**
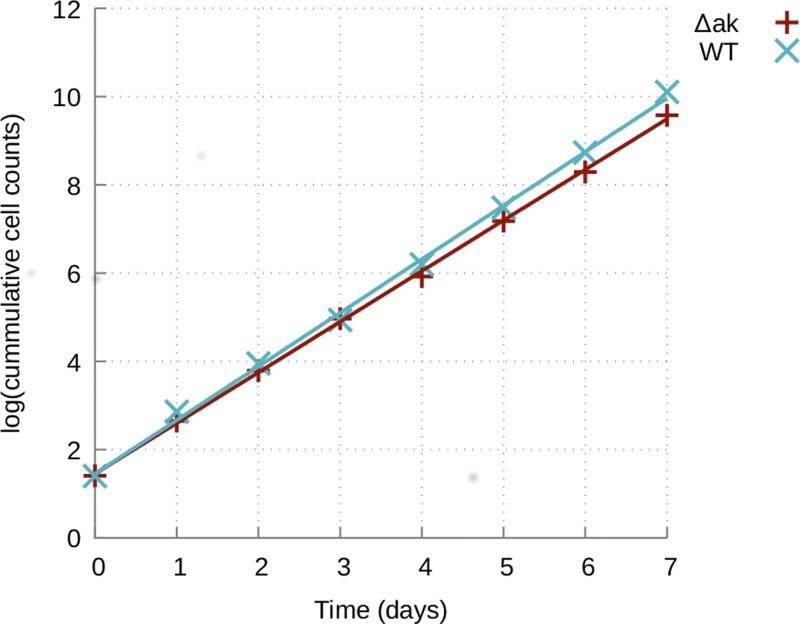
Growth of wild type and *Δak* cells

Finally, a metabolite with a mass consistent with l-arginine phosphate is also detected (37 ± 1% being 6 carbon labelled) which is consistent with the presence of arginine kinase (AK) in these cells (EC 2.7.3.3, gene Tb927.9.6290, Tb927.9.6250 and Tb927.9.6210) [60], localised to the glycosome, cytosol and flagellum, respectively [60]. In order to confirm that the arginine phosphate detected was indeed derived from AK, we knocked out all three highly similar isoforms of the gene in the BSF of *T. brucei* (see Supplementary Figure S1). The *Δak* strain was viable and had a similar growth rate to the wild type (Figure 8). In addition, untargeted metabolite profiling of the *Δak* line showed that arginine phosphate was no longer present, confirming that l-arginine is the sole source of arginine phosphate in these cells. Interestingly the level of l-arginine was also reduced by 1.7-fold, but no significant global metabolic changes were observed between *Δak* and wild type cells.

Although no significant metabolic changes were caused by the absence of AK genes, arginine phosphate is believed to play a critical role as an energy reserve by transferring the high-energy phosphate of arginine-phosphate back to ADP to form ATP when it is required quickly, for example under severe stress conditions such as starvation, nutritional and pH stress in trypanosomes [61,62].

**Figure 9:**
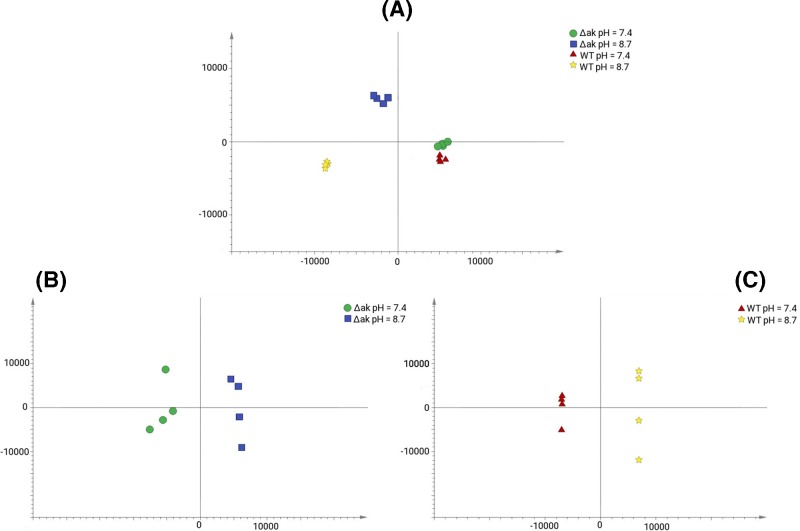
OPLS-DA scores plot of the metabolomics pH experiment (**A**) OPLS-DA including all four groups: WT and Δak cells at pH 7.4 and 8.7. (**B**) OPLS-DA including only Δak cells at both pH. (**C**) OPLS-DA including only WT cells at both pH.

The role of AK in trypanosome survival at high pH was also investigated. The survival rate of *Δak* and WT cells grown at pH 7.4 (normal growth condition) and 8.7 (high pH condition) was investigated by counting the number of cells surviving after 72 h of cell culture at each pH condition. The *Δak* cells showed a 52% decrease in survival capability at pH 8.7 compared with the cells grown at pH 7.4 (*Δak* at pH 7.4, (5.78 ± 0.25) × 10^6^ cells/ml; AK KO at pH 8.7, (3.37 ± 0.12) × 10^6^ cells/ml) whereas the WT cells showed only a 22% decrease in cell density growing at pH 8.7 (WT cells at pH 7.4, (6.27 ± 0.1) × 10^6^ cells/ml; WT cells at pH 8.7, (5.54 ± 0.47) × 10^6^ cells/ml) (Table 2). This indicates that arginine kinase improves the ability of WT cells to resist high pH stress conditions. In order to further investigate the effects of high pH growth conditions at the molecular level, extracts from the *Δak* and WT cells grown under pH 7.4 and 8.7 were analysed by LC–MS. OPLS-DA was then performed to determine specific metabolites affected by the different growth conditions. As can be seen in Figure 9A, a clear separation was observed between the normal growth and high pH growth conditions (*Δak* grown at pH 7.4 (A74) vs pH 8.7 (A87); WT grown at pH 7.4 (W74) vs pH 8.7 (W87)). Next, to identify which metabolites contributed to the separation, 2-way orthogonal comparisons were made between each cell line with the two different growth conditions (Figure 9B,C). Key metabolites contributing to the separation were selected by VIP values higher than one from all 2-way comparisons. Additionally, the selected metabolites were cross-checked by univariate *t*-test analysis (with the false discovery rate *Q* ≤ 0.05). Three metabolites are changed significantly more in *Δak* cells than in WT, all increased in pH 8.7 compared with pH 7.4, and 15 are changed significantly more in WT cells than in *Δak*, all decreased (see Supplementary Tables S2 and S3). The role of these metabolites in the response to high pH and their interactions with AK remains to be investigated.

**Table 2:** Cell counts after 2 h of growth in medium pH 7.4 or 8.7

Cell type	pH 7.4	pH 8.7	Decrease (%)
427 WT/ml (×10^6^)	6.27 ± 0.1	5.54 ± 0.47	22
*Δak* ml^−1^ (×10^6^)	5.79 ± 0.25	3.37 ± 0.12	52

### Unexpected metabolites

Using isotope labelled substrates allows detection of unexpected and novel metabolites too. For example, tracing U-^13^C glucose showed the presence of high carbon sugar phosphates such as octulose 8-phosphate and nonulose 9-phosphate as well as a number of pyruvate-derived conjugates in *T. brucei* [5]. Using U-^13^C amino acids in the experiments reported here we are able to add information on the provenance of some of the metabolites identified in the glucose labelling experiments as well as detect several new unknown metabolites (see Table 3). Creek et al. detected metabolites with masses corresponding to carboxyethyl-l-arginine and carboxyethyl-l-ornithine with 3 carbons ^13^C labels derived from glucose. Labelled arginine confirmed the presence of these two metabolites with the 6 non-glucose derived carbons of ‘carboxyethyl-l-arginine’ (or d-octopine) coming from arginine and the 5 non-glucose carbons of ‘carboxyethyl-l-ornithine’ coming from arginine via ornithine.

**Table 3:** New metabolites

Formula	Carbon labelled	Putative source
C_8_H_16_N_2_O_4_	5 from arginine, 3 from glucose	l-Ornithine + pyruvate?
C_9_H_18_N_4_O_4_	6 from arginine, 3 from glucose	l-Arginine + pyruvate?
C_8_H_11_NO_6_S	5 from glutamine, 3 from cysteine	Oxoglutarate + cysteine? Product of an aminotransferase reaction using glutamylcysteine as a substrate?
C_10_H_14_N_2_O_7_S	5 from glutamine, 3 from cysteine	C_8_H_11_NO_6_S + glycine? Product of an aminotransferase reaction using glutathion as a substrate?
C_6_H_11_NO_4_S	3 from cysteine, 3 from glucose	Cysteine + pyruvate?
C_6_H_9_NO_4_S	3 from cysteine, 3 from glucose	C_6_H_11_NO_4_S–H_2_?
C_8_H_17_NO_6_S	3 from cysteine, 5 from glucose	Cysteine + pentose?

The formula are assigned based on a measured *m/z* within 3 ppm of the calculated *m/z*. No putative identification could be made that would match mass and labelling patterns.

The metabolite with a formula C_6_H_9_NO_4_S was hypothesised to be a pyruvate-cysteine adduct [5] and the cysteine labelling experiment confirms that the 3 non-glucose derived carbons are from cysteine. A related metabolite of formula C_6_H_11_NO_4_S which has a different retention time and the same carbon labelling pattern was also found. A third metabolite containing labelled carbon derived from both glucose and cysteine of formula C_8_H_17_NO_6_S has a labelling pattern suggestive of its being formed from cysteine and a pentose, most likely ribose. Roles for these metabolites have not been assigned, and they have not been described in other systems.

Finally, we can also see two metabolites formed from 5 carbons coming from glutamine and 3 carbons from cysteine. One has a mass that matches the formula C_8_H_11_NO_6_S and could be produced from 2-oxoglutarate and cysteine, possibly the same way glutamylcysteine is produced (see Figure 2). The other has a mass that matches the formula C_10_H_14_N_2_O_7_S which could be C_8_H_11_NO_6_S used by glutathione synthetase instead of glutamylcysteine.

All these unexpected metabolites are absent from the fresh medium samples. They could be produced either by non-enzymatic reactions caused by the accumulation of particular metabolites within cells, or by enzymes using non-conventional substrates, as is the case for hydroxyglutarate (see section Glutamine). In culture, pyruvate accumulates to high concentrations inside and outside of the cells [18,35] which could explain the pyruvate derived metabolites. However, for the glutamine and cysteine derived metabolites accidental enzymatic reactions seem more likely.

## Conclusion

Here we showed how *T. brucei* bloodstream form cells metabolise five amino acids: glutamine, cysteine, proline, methionine and arginine. In addition to confirming the operation of a range of pathways, both catabolic and anabolic, we detected possible new metabolites, roles for which will need to be further investigated. Our results also indicate that, as shown in previous proteomics datasets [63], pathways are likely present in the mitochondrion that were previously ignored or downplayed, particularly for glutamine consumption. However, only a very low amount of proline is used by the parasite, indicating that the enzymes are present but only minimally active, perhaps allowing the cells to be more resistant to changes in their environment.

## Supporting information

**Supplementary Figures:** 

## Abbreviations

AdoMet: *S*-adenosylmethionine
AK: arginine kinase
CMM: Creek’s ‘Minimal’ Media
LC–MS: liquid chromatography–mass spectrometry
MTA: methyl-5′-thioadenosine
MTR: methylthioribose
NOS: nitric oxide synthase
OPLS-DA: orthogonal partial least squares-discriminant analysis
VIP: variable importance in projection values
