# Supplementary material for: Mapping the metabolism of five amino acids in bloodstream form Trypanosoma brucei using U-13C-labelled substrates and LC–MS
Source: Biosci Rep. 2019 May 17;39(5):BSR20181601. doi: 10.1042/BSR20181601 (PMC6522824; doi:10.1042/BSR20181601)

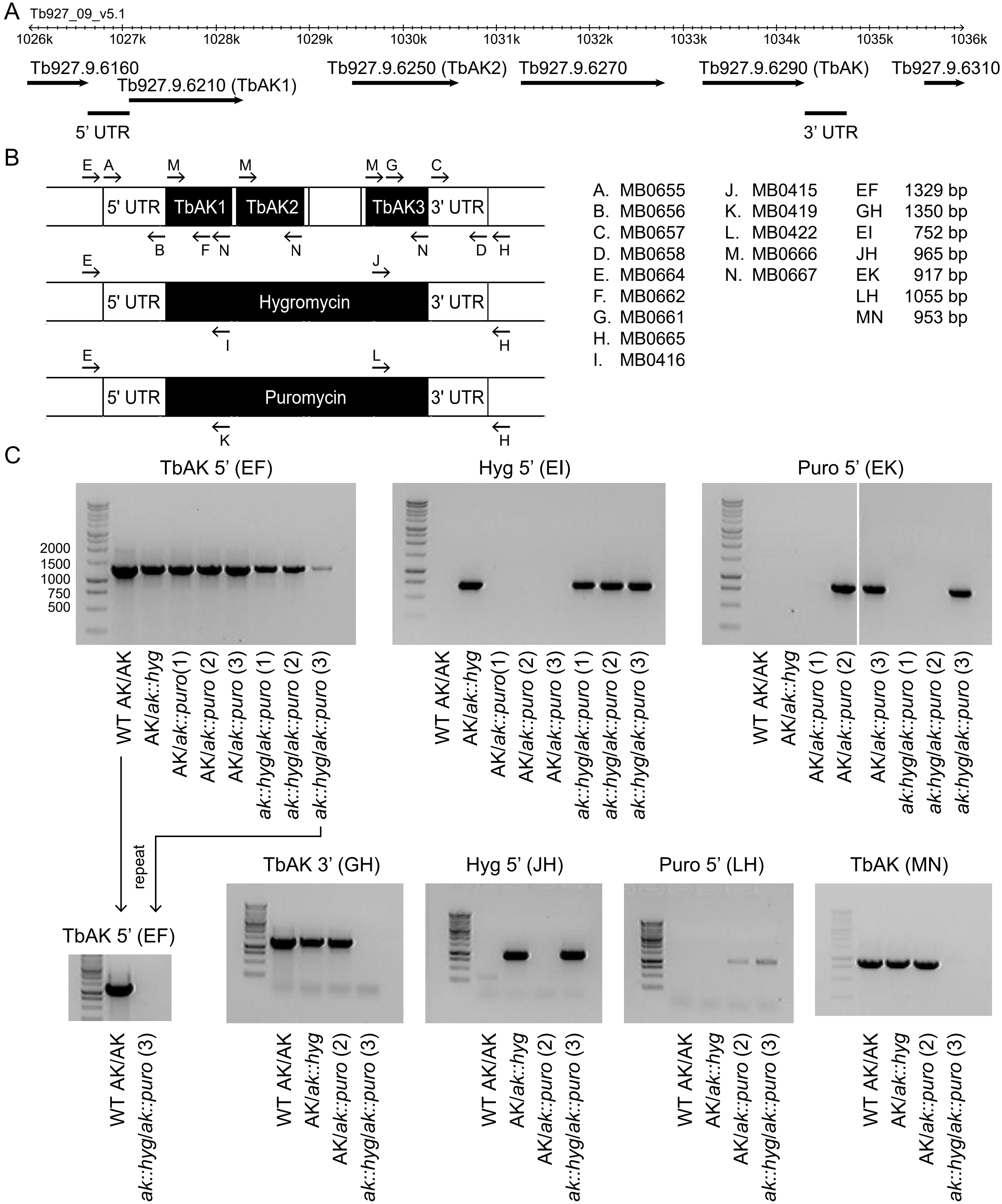
A
Tb927_09_v5.1
1026k
1027k
1028k
1029k
1030k
1031k
1032k
1033k
1034k
1035k
1036k
Tb927.9.6160
Tb927.9.6210 (TbAK1)
Tb927.9.6250 (TbAK2)
Tb927.9.6270
Tb927.9.6290 (TbAK)
Tb927.9.6310
5' UTR
3' UTR
B
5' UTR
TbAK1
TbAK2
TbAK3
3' UTR
Hygromycin
Puromycin
A. MB0655
B. MB0656
C. MB0657
D. MB0658
E. MB0664
F. MB0662
G. MB0661
H. MB0665
I. MB0416
J. MB0415
K. MB0419
L. MB0422
M. MB0666
N. MB0667
EF 1329 bp
GH 1350 bp
EI 752 bp
JH 965 bp
EK 917 bp
LH 1055 bp
MN 953 bp
C
TbAK 5' (EF)
Hyg 5' (EI)
Puro 5' (EK)
2000
1500
1000
750
500
WT AK/AK
AK/ak::hyg
AK/ak::puro(1)
AK/ak::puro (2)
AK/ak::puro (3)
ak::hyg/ak::puro (1)
ak::hyg/ak::puro (2)
ak::hyg/ak::puro (3)
repeat
TbAK 5' (EF)
TbAK 3' (GH)
Hyg 5' (JH)
Puro 5' (LH)
TbAK (MN)

LProline

Formula: C5H9NO2 Mass: 115.063 Std.RT: 575.4 Ion: NEG

G1

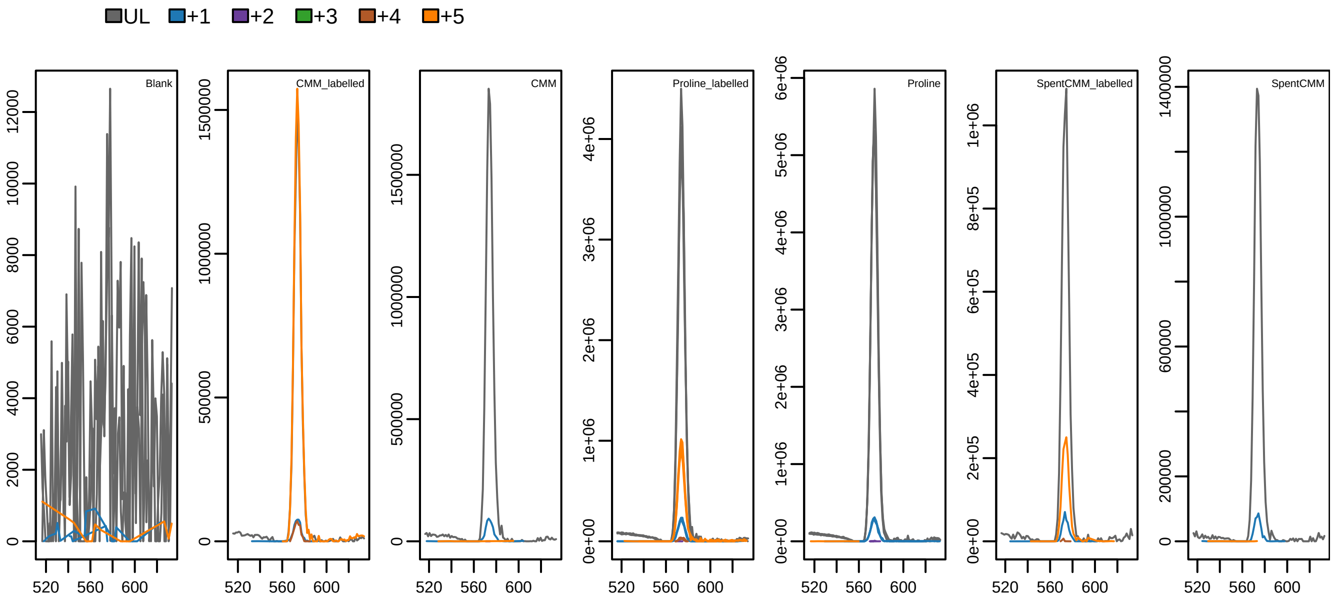

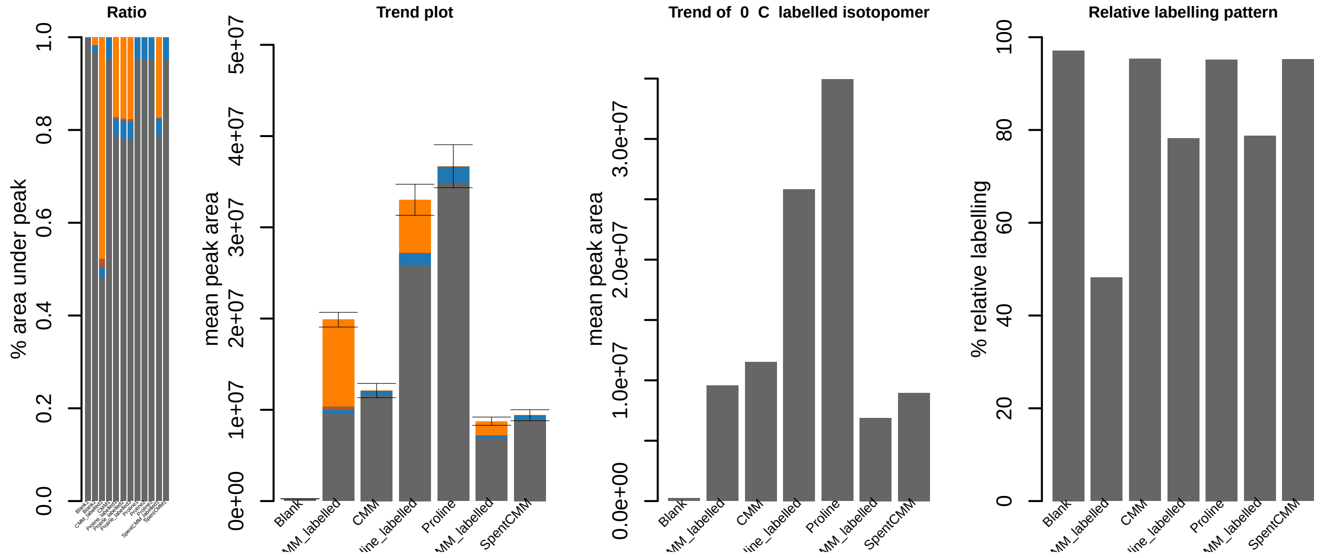

glutathione

Formula: C10H17N3O6S   Mass: 307.084 Std.RT: NA Ion: NEG

G1

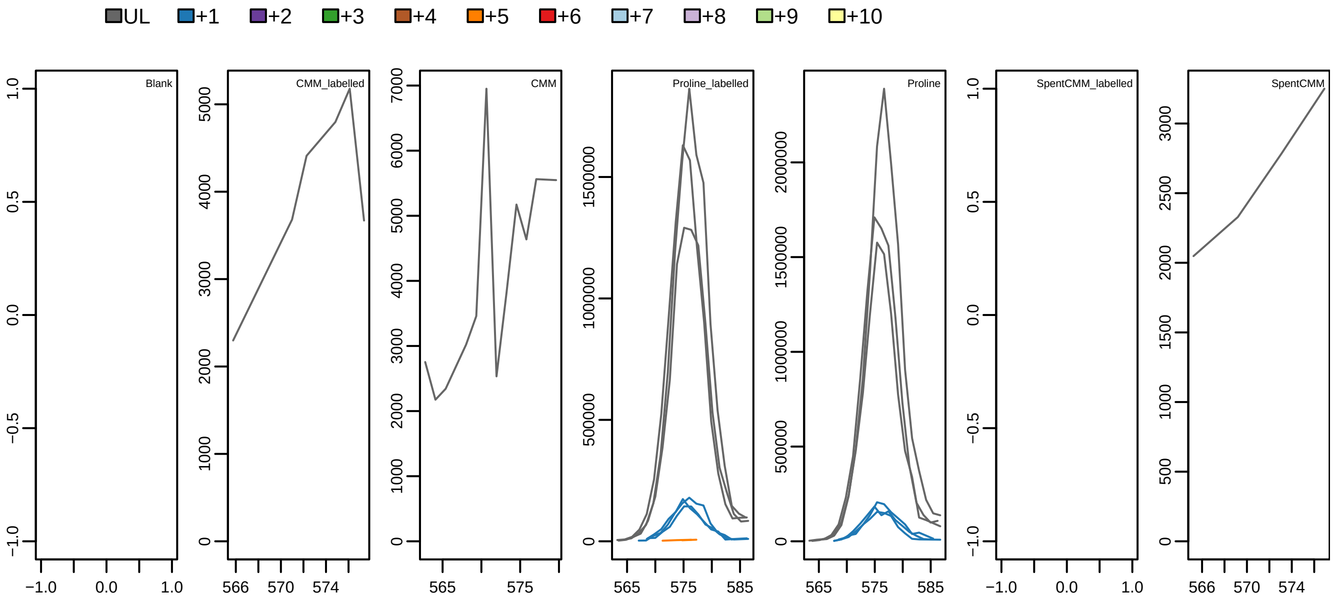

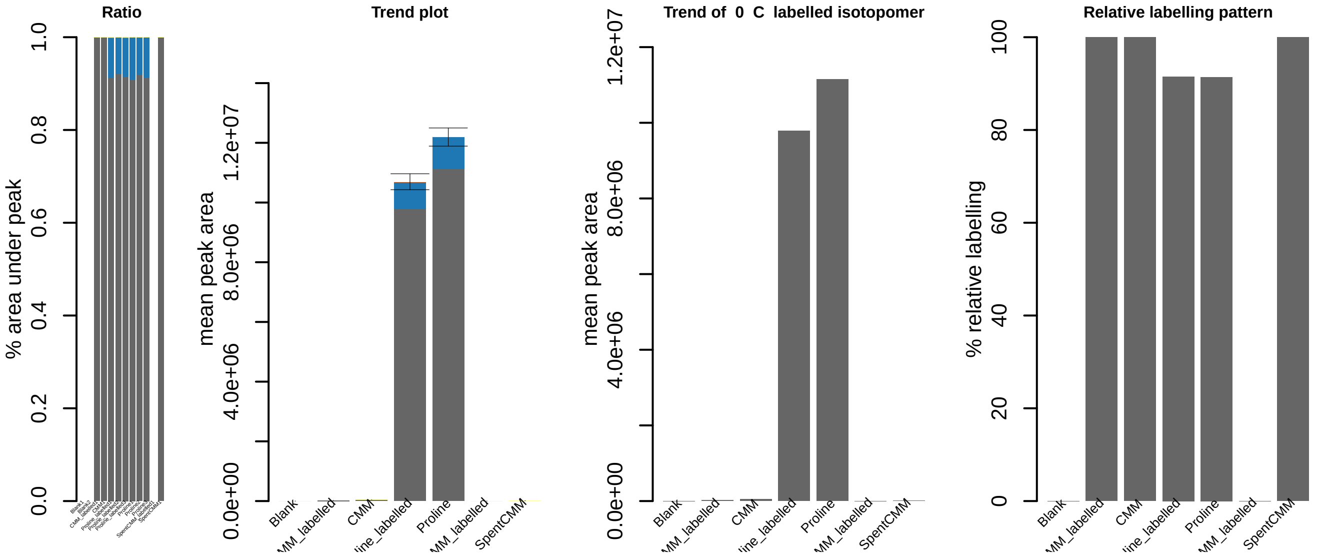

(R)2Hydroxyglutarate

Formula: C5H8O5 Mass: 148.037 Std.RT: 606 Ion: NEG

G1

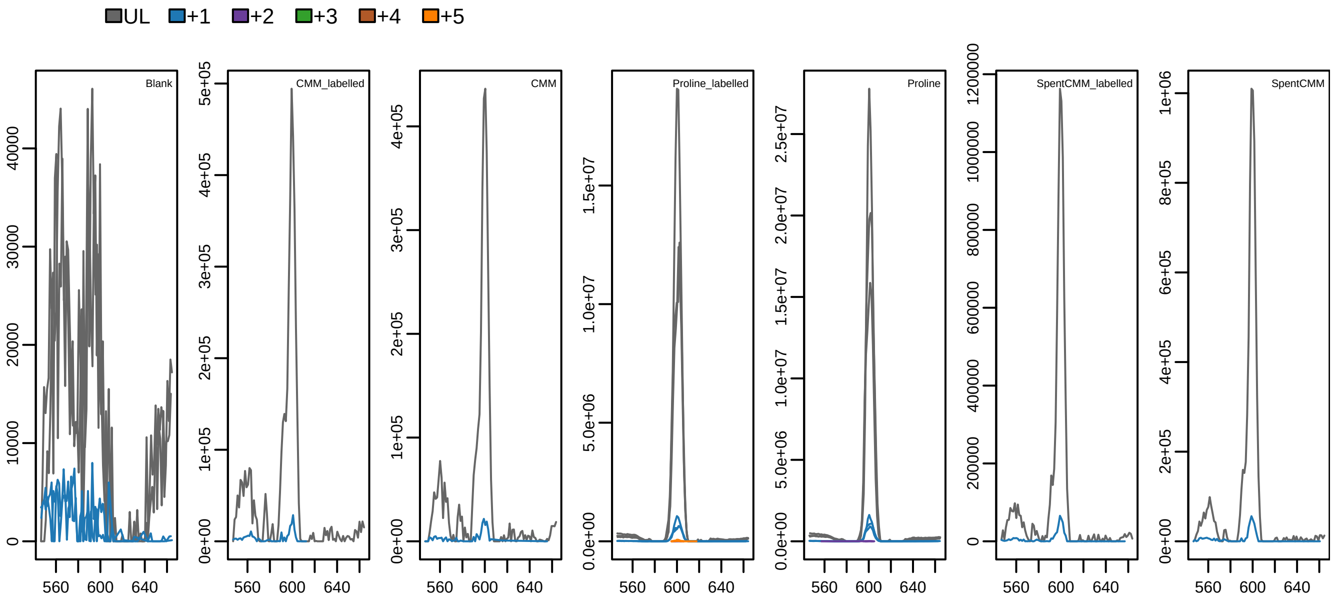

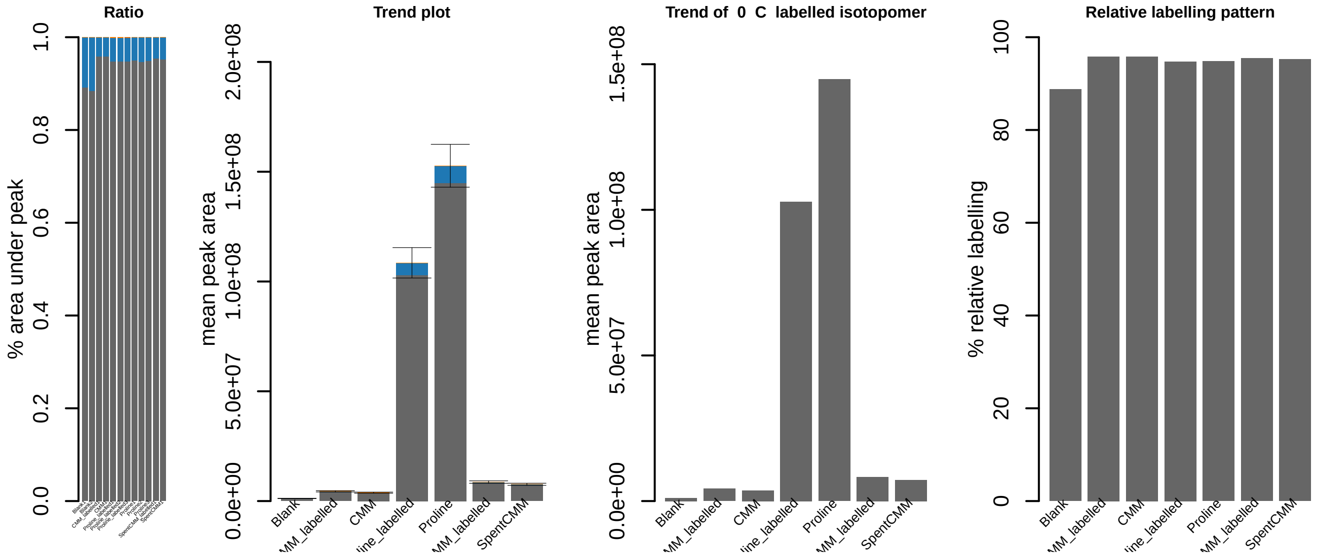

Succinate

Formula: $C_4H_6O_4$ Mass: 118.027 Std.RT: 604.2 Ion: NEG

G1

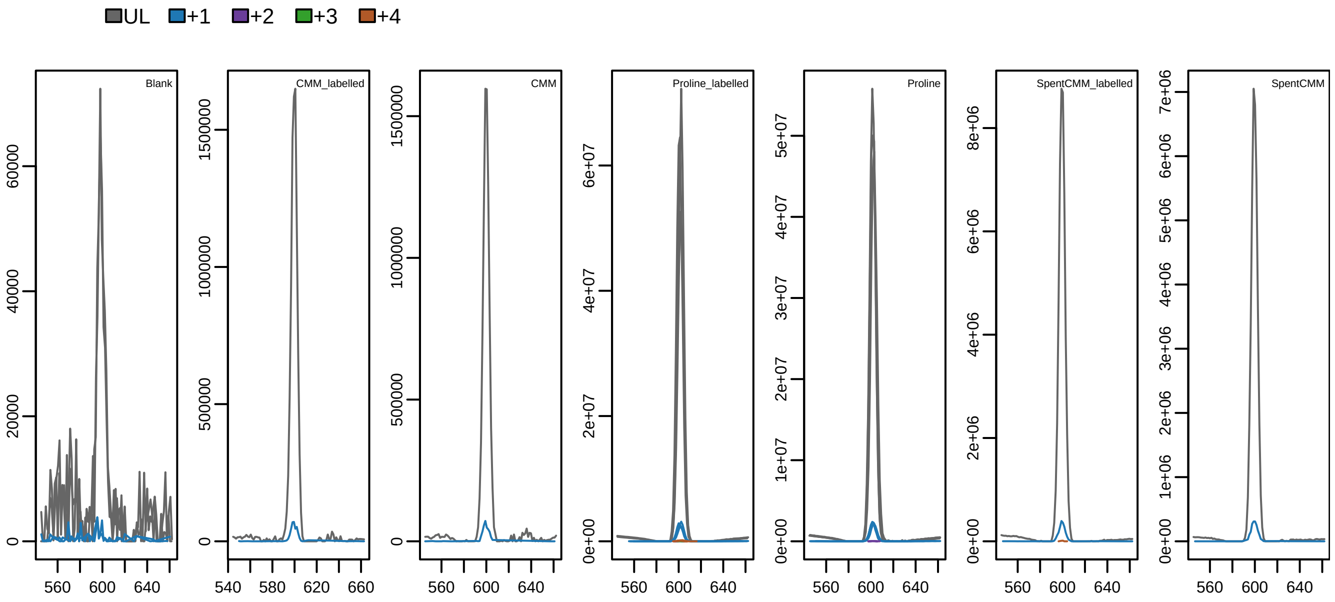

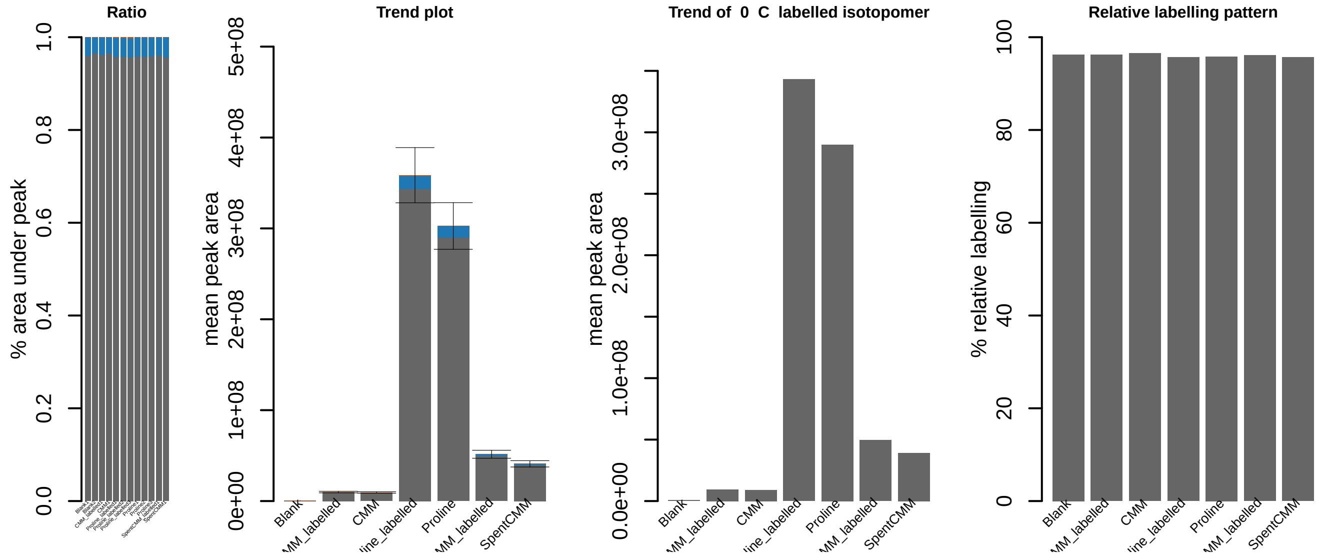

Trypanothione disulfide

Formula: C27H47N9O10S2   Mass: 721.289 Std.RT: NA Ion: NEG

G1

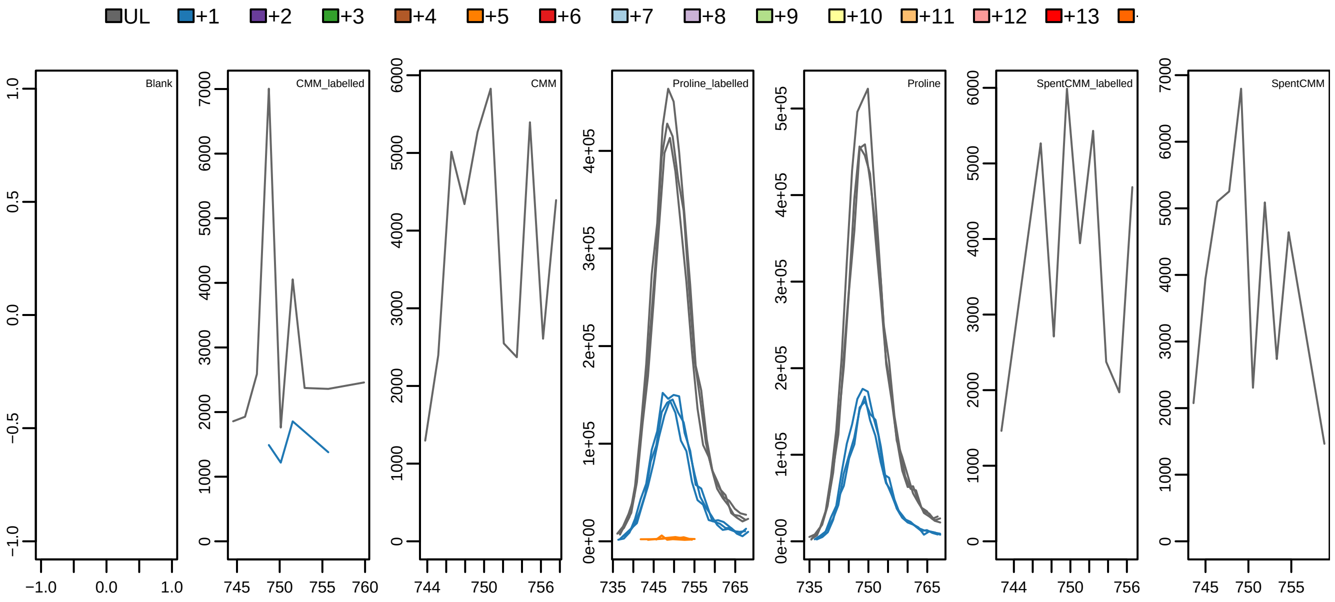

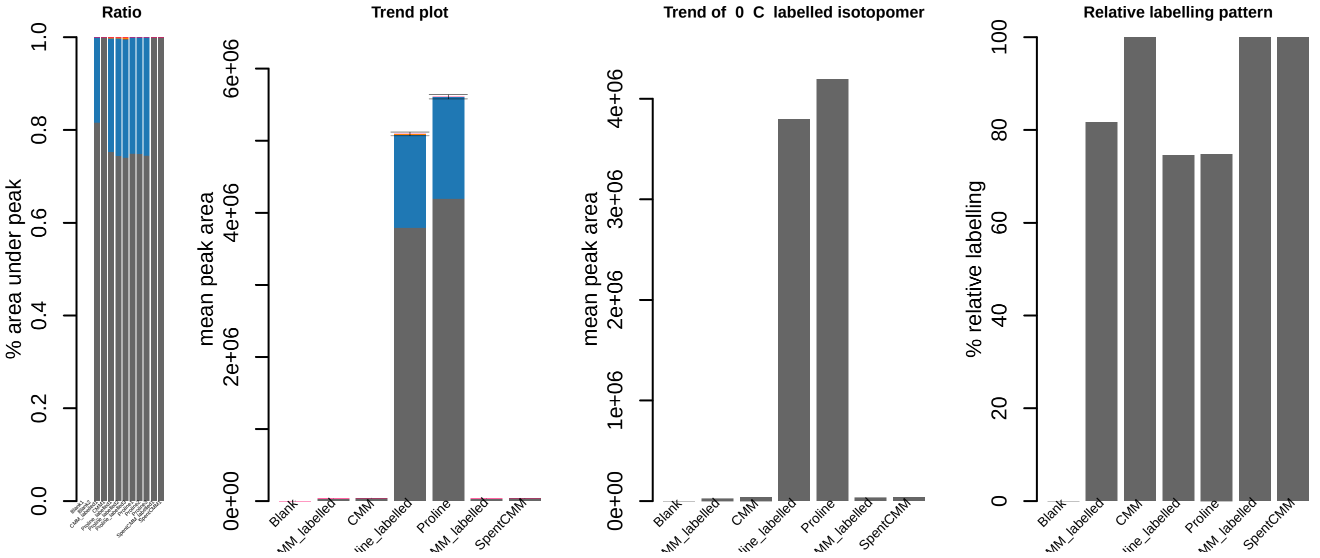

Supplement: Supplementary file 1 [file bsr20181601_Supp1.pdf]
